# Different ways to repair the mitral valve with artificial chordae: a systematic review

**DOI:** 10.1186/1749-8090-5-22

**Published:** 2010-04-08

**Authors:** Federico Bizzarri, Antonella Tudisco, Massimo Ricci, David Rose, Giacomo Frati

**Affiliations:** 1 Cardiac Surgery Unit, Polo Pontino, Heart and Great Vessels Department, University of Rome "Sapienza", Latina, Italy

## Abstract

Myxomatous mitral regurgitation (type II Carpentier's functional classification) affects about 1-2% of the population. This represents a very common indication for valve surgery resulting in a low percentage of repairs compared to replacement which is actually performed. In the last decades, several methods for mitral valve repair have been developed, to make the surgical feasibility easier, improve the long-term follow-up thus avoiding the need for reoperations. A very interesting method is represented by the combination of various valve repair techniques, depending on the involvement of the anterior, posterior, or both leaflets, and the use of PTFE artificial chordae tendineae when excessive chordal elongation or rupture due to myxomatous degeneration co-exists. The aim of this review is to summarize the evolution of these techniques from the beginning till now.

## Materials and methods

We performed MEDLINE and bibliographic search including 27 articles published between 1984 and 2009 regarding the different applications of mitral valve repair with implantation of artificial PTFE chordae tendinae. The key words we used were mitral, repair, artificial chordae. Most of the techniques we analyzed were employed to repair both leaflets. Atriotomy approach is performed in all but one technique, in which an aortotomy is made too. The main difference between the techniques is in the measurement of the length of the artificial chordae. The oldest and most common method to calibrate the length of the neo-chordae consists in filling the left ventricular cavity with saline solution. Other authors elongated the prosthetic chordae trying to approximate the coaptation area between the two mitral leaflets. Recently, a variety of different calipers that allow in some manner to check the length and to tighten the number of necessary chordae have been introduced to better define the adequacy of the PTFE chordae implantation. One group uses intraoperative transesophageal echocardiography to measure the necessary length of the chordae. Scorsin et al. and Smith et al. used new devices with premeasured artificial chordae. Maselli et al. proposed a method for "tuning" the lenght of the artificial chordae during the operative time. The most interesting and forward-looking technique we analyzed was the one proposed by Smith et al.

## Technique Details

**Morita and colleagues **were the first to use 4-0 PTFE figure of 8 to repair both leaflets prolapse passing from the papillary body to the leaflet and back adding a Kay annuloplasty at the end of the procedure [1].

**Zussa**, one of the pioneers of this technique, repaired an anterior leaflet with PTFE strings passing through the head of the papillary muscle and tying over a reinforcing autologous pericardial pledget. The strings were then anchored to the free margin of the anterior mitral leaflet at the unsupported areas and reinforced with a small autologous pericardial pledget. The two strands were tied after filling the ventricular cavity with saline solution for adjusting the chordal length [2].

**Murakami and colleagues **approached the anterior leaflet prolapse using mattress e-PTFE suture with Teflon or autologous pledget passed through the free margin of the leaflet from the ventricular side to the atrial side. The two arms of the suture, reinforced with pledgets, were brought down to the papillary muscle and passed through it. The length of the e-PTFE chordae was then adjusted by approximating the coapting area of the opposite leaflet and the ends of the sutures were then tied together [3].

Chordae tendinae reconstruction, in patients with prolapse of anterior leaflet was done by **Matsumoto and colleagues **in children using the following technique: double-armed mattress e-PTFE sutures were passed through the free prolapsed edge from the ventricular side to the atrial side and then the two ends were passed through the papillary muscle at 3 to 4 mm from its top, drawing the free edge down to the entry point on the papillary muscle of the two ends of the suture. The sutures were passed through a pledget, which would be on the side where the sutures emerged from the papillary muscle. The knot was tied at the level of the opposing normal leaflet. The new chorda was pulled back through the papillary muscle until the pledget came up against the muscle. Another e-PTFE suture was placed in the same fashion. A Kay-Reed annuloplasty was added [4].

**Kawahira and colleagues **used 4-0 e-PTFE sutures through the prolapsed leaflet from its ventricular to atrial aspect, placing pledgets for reinforcement on the ventricular surface of the leaflet. The sutures were anchored to the papillary muscles in a mattress fashion. This maneuver could be carried out in reverse order: attaching e-PTFE suture initially to the papillary muscle, subsequently passing it through the leaflet from it's ventricular to atrial aspect. In this circumstance, the knot would be placed on the atrial aspect of the mitral valve [5].

**Adams and colleagues **placed one or more 4-0 Gore-Tex sutures into the head of the papillary muscle. Papillary muscle exposure was enhanced after quadrangular posterior leaflet resection. Before annuloplasty poor leaflet apposition is present in all leaflet segments with saline testing and segmental anterior leaflet prolapse is best identified by height comparison with a normal reference point. After ring annuloplasty symmetric leaflet apposition limits leaflet incompetence to the prolapsing anterior leaflet segment. Both arms of the previously placed Gore-Tex suture are passed through the margin of the prolapsing leaflet segment. Passing the suture through the free edge of the cusp twice as well as starting with a surgeon's knot are techniques to prevent overaggressive sliding of the knots when tying the Gore-Tex suture [6].

**Tomita **applied the method of David [7] to use the reconstruction of the valve with CV-4 e-PTFE sutures. The double armed suture is passed twice through the fibrous portion of the papillary muscle head that anchors the elongated or ruptured chordae and is tied down (seven or eight knots are needed for this type of suture material). The two arms of the suture are then brought up to the free margin of the leaflet and passed through the point where the original chorda was attached (thickened portion of the leaflet). The needle is brought from the ventricular side of the leaflet to its atrial side and then passed once through the leaflet. The length of the PTFE chordae is adjusted by approving the coating area of the opposite leaflet. Then both ends of the suture are passed through the leaflet again and tied together on the ventricular side. Another PTFE suture is placed when the prolapsed portion is wide. Kay's annuloplasty is added at the end [8].

With time, it appeared mandatory to find the correct technique to determine the length of the artificial chordae.

**Sarsam and colleagues **passed one or more 5-0 e-PTFE sutures, supported by a felt pledget through the fibrous portion of the papillary muscle. The suture was left untied. The two arms of the suture were then passed once through the rough free edge of the prolapsing leaflet from the ventricular to the atrial side. If the native chorda to the corresponding part of the opposing leaflet are normal, the edges of the anterior and posterior leaflet are temporarily approximated by a simple or figure 8 suture and then the suture is tied against the temporary suture. Three knots are used. The suture in passed again through the edge of the leaflet from the ventricular to the atrial side and tied permanently. The temporary suture is then removed [9].

**Soga **made a resection of both the anterior and posterior mitral leaflets and subvalvular apparatus and placed two 3-0 e-PTFE mattress sutures: one placed and tied at the tip of the anterior papillary muscle, and one at the tip of the posterior papillary muscle. The suture of the anterior PM is placed at the 9-10 o'clock position on the mitral annulus (as defined by mid-anterior annulus to be 0 o'clock), and the suture for the posterior PM at the 5-6 o'clock. According to the authors, the length of the artificial CT can be determined during intraoperative cardiac arrest, and may be suitable if the sutures are tied just less than taut before insertion of the prosthetic. After the valve replacement, the motion of the prosthetic leaflets is examined to ensure that the leaflet are not entrapped by the 3-0 e-PTFE sutures [10]

**Tomita **repaired chordae tendinae with CV-4 e-PTFE sutures. Double armed sutures are passed twice through the fibrous portion of the PM head that anchors the elongated or ruptured chordae and are tied down (7 or 8 knots are needed for this suture material). The two arms of the suture are brought up to the free margin of the leaflet and passed through the point where the original chorda was attached (thickened portion of the leaflet). The needle is brought from the ventricular side of the leaflet to its atrial side and passed once more through the leaflet. The length of PTFE chordae is adjusted by referring the contact area of the opposite leaflet and then both ends of the suture are passed through the leaflet again and tied together on the ventricular side. When the prolapsed portion became wide, another PTFE suture was placed in the same fashion. At the end Kay's suture annuloplasty (n = 24) or ring annuloplasty [11] was performed.

**Minami **used double armed mattress sutures of 4-0, 5-0 or 6-0 e-PTFE placed to reinforce with felt pledgets between the PM and free margin of the anterior leaflet. The length of the PTFE sutures was adjusted with the adjacent normal anterior leaflet or facing posterior leaflet. When the prolapsed portion became wide, another suture was placed in the same fashion. The number of sutures ranged from 1 to 3. In addition, Kay annuloplasty was perfomed [12].

**Matsui **employed a new device (Matsuda Ika-Kogyo, Tokyo, Japan) consisting of two metallic tubes with a circular, hook shaped distal tip made entirely of stainless steel. The distal tip, which is perpendicularly attached to the inner tube, was designed to hold the Gore-Tex thread at the reference point on the PM immovable. The outer tube could slide on the surface of the inner tube to measure the length from the tip of inner tube to the hook of outer tube. A 4-0 or 5-0 Gore-Tex mattress suture, reinforced with a felt pledget, was placed into the head of the PM. Both arms of the suture where left untied. Length was determined by measuring the distance between the leaflet edge and the site of implantation of the artificial chordae on the PM, using a normal valve segment adjacent to the prolapsing segment as a reference. The distal tip of the inner tube of the device was placed at the sutured site of the artificial chordae on the PM. The proximal hook of the outer tube was slid to the edge of the adjacent non prolapsing leaflet and then fixed at that point after reading the distance between the distal tip and proximal hook to the device. Devices were then moved to the prolapsed segments so as to hold an edge of the prolapsed leaflet with a proximal hook. As the determined distance and edge of the leaflet were fixed with the device, the Gore-Tex suture could be tied in the usual manner without knot slipping. The action of knot-tying itself works to immobilize the device by its strength. After removing the device, followed by saline testing, a Carpenter-Edwards annuloplasty ring was attached according to the size of the mitral annulus [13].

**Prêtre and colleagues **applied the artificial chordae to the mitral valve using an approach through the aortic valve for an anterior and posterior leaflet prolaps. In the anterior repair an atriotomy was performed first, the artificial chordae was placed in the usual manner, and then a flexible annular ring was tied on the mitral annulus. An aortotomy was performed to expose the native chordae and to calibrate the length of the artificial chordae that were locked but not tied down. The mitral valve was inspected through the atriotomy while saline water was injected through the aortotomy in the left ventricle. The chordae were tied from the aortotomy and the incisions closed in the usual fashion. In the posterior leaflet prolapse, repair was done and a ring was inserted using a classical atrial incision. The ascending aorta was opened and the artificial chordae were set on the papillary muscles and the anterior leaflet was calibrated. The valve was re-inspected through the atriotomy with instillation of saline in the left ventricle for adjusting the chordae until they were definitively secured [14].

**Lawrie and co-authors **published their experience on 152 consecutive patients. 5-0 PTFE sutures were placed into the bases of the papillary muscles in a figure-8 fashion, and were brought through the free edge of the prolapsing segment. Dots were made to mark the desired final line of leaflet apposition. The left ventricle was inflated with saline solution and the chordal length was adjusted to align the edges of the leaflets. Leaflet alignment was checked and the PTFE was tied down. The knot was locked with a 6-0 polypropylene stitch which was tied over the end of the PTFE to prevent sliding of the PTFE knots. An annuloplasty ring was then implanted [15].

**Calafiore **in the anterior leaflet prolapse passed 4-0 PTFE sutures through the fibrous tip of the papillary muscle and fixed the sutures. The new chorda was passed in the border of the anterior leaflet in the proper place and its final length was measured with a ruler. A mark was applied to indicate this distance and the suture was tied with the aid of a nerve hook [16].

**Rankin **in the anterior and/or posterior leaflet prolapse placed 4-0 prolene pledgetted horizontal mattress sutures longitudinally into each papillary muscle, passing one arm through the fibrous tip, and tying firmly; through this anchor suture, a double-armed Gortex suture was passed but not tied. A Carpentier annuloplasty ring was sutured. With the ring in position, the chordae were retrieved from the ventricle, and both needles were woven into the prolapsing segment, straddling the point of maximal prolapse. Two or three bites were taken through the coaptation surface to the line of coaptation. The two arms of the suture were tied on the atrial surface with a slip-knot to bring the leaflet to the annular plane, and a clip was placed across the knot. Pericardial pledgets could be used if the leaflet tissue seems fragile. Cold saline solution was infused to check the length of the suture; once the valve was competent, eight more knot were tied tightly against the clip, the suture was cut, and the clip was removed [17].

**Tam **used the following technique for any prolapsing segment. A calliper was used to measure the length of the reference chordae. A 4-0 ePTFE suture was used to create loops around the calliper. Non-sliding knots were placed at the end of each loop while still on the calliper. After making a desired number of loops, the needles were passed through the loops and tied. Two needles at the end of the sutures were passed through an ePTFE pledget, which was now ready to be secured to the papillary muscle. The ePTFE chordae were secured at the tip of the papillary muscle with two pledgets and attached to the edge of the prolapsing mitral leaflet using eight 5-0 ePTFE sutures [18].

**Mandegar **for any leaflet prolapse used following technique. During preoperative transesophageal echocardiography, a line was drawn between the base of the anterior and posterior mitral leaflet to measure the distance between the head of the posterior papillary muscle and the plane at the co-optation of the leaflets; this measured the artificial chordal length. During surgery, 4-0 Gore-Tex was passed through the fibrous tip of the papillary muscle with a pledget and was fixed with a loose knot. Two tight reverse knots were made for every millimeter of 4-0 Gore-Tex that was required. The needles were passed through the edge of the anterior leaflet at the prolapsing portion, and the Gore-Tex was knotted onto a strip of pericardium so that the final knot could be placed at the atrial side of the leaflet [19]

**Gillinov **describe a technique for reparing anterior leaflet prolapse. Chordal length was determined with a calliper, and ePTFE chordae were constructed making loops around it. A pledget was used to prepare the number of 5-0 ePTFE loops that were needed. When all chordal loops were constructed, each needle was passed through the head of the papillary muscle, and was affixed to the free edge of the anterior leaflet with a figure 8 suture of CV-5 ePTFE [20]

**Scorsin**: any leaflet prolapse. Artificial chordae system device was composed of 2 sets of 4 artificial chordae, attached to a 3-mm strip of knitted polyester 18 mm wide, leaving 4 mm between each chorda. The device was applied by suturing the strip to the free edge of the prolapsed leaflet by continuous suture. Each array was anchored to the tip of the correspondent papillary muscle by only one stitch. After this procedure, a prosthetic annuloplasty ring was inserted [21].

**Maselli and De Paulis **used a novel system to repair the valve consisting of two components: leaflet component and the papillary component. The first one was achieved with a CV-5 PTFE suture. A circular loop was obtained at the middle of the suture by tying it around a Hegar dilator with a diameter of 13 mm. Flattened loop's length should equals half the circumference. Given a circumference of approximately 4 cm for a circle with a diameter of 13 mm the length of the loop would be approximately 2 cm. Papillary component was obtained by cutting a CV-4 PTFE suture in 2 halves; 5 double knots were placed at a distance of 2 mm at the needleless tip of each CV-4 semisuture. The needleless tip of the suture was anchored on a drape; knots were placed with the help of forceps and a needle holder and slid into definitive position by inserting the tip of the needle or a nerve hook in the knot itself. To realize papillary component for each neochorda 2 CV-4 half sutures with knots were needed. After the assessment of the mitral valve lesions, the papillary component was set in place by first fixing 2 semisutures to a papillary head and tying the sutures so that the papillary head was "sandwiched" between 2 e-PTFE pledgets to reduce trauma. Two CV-5 loops were fixed on the desired leaflet 2 to 3 mm apart from the leaflet's edge, passing the needle from the atrial to the ventricular side and leaving knots on the ventricular aspect of the leaflet. A single PTFE pledget was interposed on the atrial side. To obtain reversible coupling of the leaflet component with the papillary, a loop which could be tightened and loosened as many times as required, was placed in the leaflet component with the help of forceps and a curved instrument. The papillary component passed inside the loop and the loop was tightened. The loop had to fall in the gap between two knots. Chordal length was fixed by closing the loop under the selected reference knot of the papillary component. Same steps were repeated for the other chordae. To shorten or elongate the neochorda without touching its papillary or leaflet anchoring, the loop was released and slid under a reference knot respectively closer or farther from the papillary muscle tip, and tightened again [22].

**Boon and colleagues **used CV-5 e-PTFE sutures for older children, while CV-7 was typically used in neonates and small infants. The suture was first tied to the fibrous tip of the PM and the two ends were fixed to the free edge of the valve leaflet in a V-shape. For the anterior leaflet, a new chord length was measured by bringing the free edge of the valve to the level of the anterior annulus. The length could also be compared to healthy non-elongated native chords in the adjacent area. Then both ends of the sutures would be passed again through the free edge and tied on the ventricular side of the leaflet, to prevent the knot from interfering with the co-optation zone. Because the sutures are placed in a V-shape, one suture accounts for 2 new artificial chords. In addition, ring annuloplasty or Wooler-Kay bilateral commissural plication annuloplasty was performed [23].

**Chan**: for anterior leaflet prolapse a 4-0 Gore-Tex suture with pledgets was used. The suture was first passed through the papillary muscle and secured with 6 to 8 knots. Both braids were then passed through the prolapsed leaflet edge no more than 4 mm apart. The suture was then tensed up. The non-prolapsing posterior leaflet was used to check the reference length. A single-arm rubber-protected artery forceps was clipped on the mark, and knots were tied on it [24].

**Salvador**: for anterior leaflet prolapse repair a e-PTFE double-armed suture (GORE-TEX CV-5) were passed through the PM with a mattress technique and reinforced with autologous pericardial pledgets (rarely, GORE-TEX pledgets), on both sides of the muscle. Each end of the suture were fixed to the free margin of the prolapsed leaflet and reinforced with a small autologous pericardial pledget or a small GORE-TEX pledget. The length of the artificial chordae was adjusted to maintain the corresponding free margin of the leaflet at the desired level in the ventricular cavity. To determine the correct length of the artificial chordae, the neochordae were tied at the end of all the other repair procedures after the ventricular cavity is filled with saline solution [25].

**Smith and Stein **made the first endoscopic placement of multiple pre-measured artificial chordae with Robotic assistance and nitinol clip fixation. Robotic bileaflet mitral valve repair used a more lateral approach and 5 right thoracoscopic ports, ranging in size from 8 to 20 mm. Left atriotomy was perform to expose mitral valve using a robotically controlled EndoWrist atrial retractor (Intuitive Surgical Inc.). The prolapsing segment was identified with valve hooks. The "ski-tip" style ends of the robotic retractor blades are longed into the anterior leaflet, then the atrial septum is lifted to visualize PM. The length of the artificial chordae loops were determined with the measure of the distance between the correct plane of apposition on an adjacent normal non-prolapsing segment of the mitral leaflet and the respective PM (done with a More Suture Ruler device). Artificial chordae, with 4 loops each, were constructed of 4-0 PTFE GORE-TEX per the technique by von Oppel and Mohr. A single felt pledget constructed the platform with multiple neochords of definite length extending from its base. Both free suture needles from the pledget platform were passed through the respective PM with 2 robotic large needle drivers. After the correct placement in the muscle head, the needles were retrieved and the neochordae platform was secured with extracorporeal knots tied by the assistant using a closed knot pusher. Each neochordae loop was attached to the edge of the prolapsing leaflet by applying a single-armed V60 U-clip per loop. The single-armed U-clip was placed in the leaflet edge with a robotic large needle holder and the neochordae loop was captured in the open clip circle. The U-clip was deployed by pulling the needle off the clip portion, securing the neochordae loop to the leaflet. Additional reduction of the leaflet height could be achieved by folding the leaflet edge toward the ventricle before deploying the U-clip. The remaining loops were distributed at equal distance along the edge of the prolapsed segment by applying the same technique. After the pledget platform was secured, the 2 free suture needles were placed through the anterior prolapse. The correct apposition was confirmed with saline test. The assistan, at the patient side, tied the knots. Annuloplasty was performed at the surgeon's discretion. For concomitant left atrial ablation a SurgiFlex XL probe was applied endocardially. Lastly the heart was de-aired and the left atrium was closed with a running suture line [26].

**Doi **measured the length of the chordae of the posterior leaflet, opposing the prolapsing portion of the anterior leaflet by TEE. The length of chordae was a measurement of the distance between the head of the PM and the free edge of the posterior leaflet. Length of the opposing chordae of the posterior leaflet was measured directly by using a calliper. Double-armed mattress sutures with CV-5 GORE-TEX were placed at the fibrous tip of the PM using PTFE on both sides and tied down firmly. In all cases Doi performed Duran ring annuloplasty. Thereafter, the ePTFE suture is placed through the anterior leaflet. The needles were passed through the rough zone of the prolapsing portion from the atrial to the ventricular side, and again through the free margin of the leaflet from the ventricular to the atrial side. The calliper that was fixed at the length of the opposing chordae was inserted inside the loop created by the ePTFE suture. The suture was easily tied at the exact length of the opposing chordae and the anterior leaflet was fixed at the height of the posterior leaflet [27].

## Conclusions

The results of these techniques we described above have been shown to be safe and effective with low rates of post operative complications or death. Robotics procedures are not widely used because of the high costs and high requirement of technique skills, but they promise to be the overwhelming choice in the very near future.

Mitral valve repair is a challenging technique deserving continuous attention over time. In the future we are waiting for more novel procedures to ensure better results in mid and long term morbidity.

## Competing interests

The authors declare that they have no competing interests.

## Authors' contributions

FB conceived the study and revised the manuscript; AT, MR and DR collected bibliographic pages references, GF revised the final manuscript before publication and checked for any typographical errors.

All authors have read and approved the final manuscript.
